# Tai Chi for improving balance and reducing falls: A protocol of systematic review and meta-analysis

**DOI:** 10.1097/MD.0000000000015225

**Published:** 2019-04-26

**Authors:** Dongling Zhong, Qiwei Xiao, Mingxing He, Yuxi Li, Jing Ye, Hui Zheng, Lina Xia, Chi Zhang, Fanrong Liang, Juan Li, Rongjiang Jin

**Affiliations:** a School of Health Preservation and Rehabilitation; b School of Acupuncture-Moxibustion and Tuina/The Third Affiliated Hospital, Chengdu University of Traditional Chinese Medicine, Chengdu, Sichuan, China.

**Keywords:** balance, falls, meta-analysis, protocol, randomized controlled trials, Tai Chi

## Abstract

**Introduction:**

To investigate the effectiveness and safety of Tai Chi for improving balance and reducing falls on people.

**Methods and analysis:**

The following databases will be searched: China Biology Medicine (CBM), China National Knowledge infrastructure (CNKI), Wan Fang Data, the Chinese Science and Technology Periodical Database (VIP), Medline, EMBASE, Web of Science, The Cochrane Library from inception to March 2019. All randomized controlled trials (RCTs) utilized Tai Chi to improve balance ability and reduce falls will be included. Primary outcomes are the fall-related indicators, including the number of falls, fall rate, and other fall-related outcomes. Additional outcomes include the Berg Balance Scale (BBS), standing-walk test, single-legged time, or other balance-related outcomes. Study selection, data extraction, and quality assessment will be performed independently by 2 reviewers. Assessment of risk of bias and data synthesis will be performed using Review Manager V5.3 software.

**Ethics and dissemination:**

The findings of this systematic review will be disseminated through peer-reviewed publication or conference presentations.

**Trial registration number** PROSPERO CRD42019127810

## Abstract 2

Supplemental Digital Content is available in the text

## 1 Introduction

Falls, as a major public health problem, is affecting people around the world. Some injuries like soft tissue injury, joint dislocation, fracture, craniocerebral injury, and so on can occur on people due to falls.^[1]^ A large-scale survey of 1,169,593 individuals reported that 42,259 (3.6%) investigators had experienced at least 1 fall injury in the 6 months preceding the survey.^[2]^ Serious fall can also lead to disability or even death.^[3]^ China's annual medical expenses caused by falls exceed 5 billion yuan, resulting in a direct or indirect social cost of approximately 160 to 80 billion yuan.^[4]^ Apparently, falls not only cause damages among people's health but also bring economic burden to society. Therefore, many efforts were made to improve this public health problem.

At present, methods like balance training, supplementation of vitamin D and calcium tablets, reduction of psychotropic medication, professional fall assessment, and education are commonly used for preventing falls.^[5–7]^

As a Chinese traditional exercise, Tai Chi not only prevails in China but also has a large number of trainers in Korea, Japan, Europe, and the United States. Tai Chi can be seen as a way of balance training in which movements are soft and gentle. The training is not limited by venues and is easy to practice. Tai Chi needs mental concentration, physical balance and coordination, muscle relaxation, and relaxed breathing, which has been widely used as an adjuvant therapy in the medical and physical fitness fields. A large number of evidence have demonstrated that Tai Chi has a positive effect in improving the balance function and promoting limb function in stroke patients.^[8–10]^ Tai Chi can also improve the function of knee joint and alleviate pain in knee osteoarthritis patients.^[11]^ Meanwhile, Tai Chi can not only improve the ability of balance control, flexibility but also has a positive effect on the cardiovascular fitness.^[12]^ In recent years, Tai Chi gradually attracted attention of the public and has been used as a method to improve balance and prevent falls for both healthy and unhealthy people.^[13–22]^

There were many randomized controlled trials (RCTs) using Tai Chi to improve balance function of people, and the results showed that Tai Chi did have a positive effect on it. So far, we did a search of related SRs and retrieved 18 published meta-analyses.^[23–40]^ We used A Measurement Tool to Assess systematic Reviews 2.0 (AMSTAR 2.0)^[41]^ to evaluate the quality of 18 included meta-analyses. The results of AMSTAR 2.0 showed that 17 SRs were considered critically low methodological quality and 1 SR was considered low methodological quality, which suggested that a higher level of evidence is needed to clarify the effectiveness of Tai Chi for balance ability in both healthy and unhealthy people. Therefore, this SR aims to evaluate the effectiveness and safety of Tai Chi for improving balance and reducing falls. We will conduct this SR and meta-analysis strictly in accordance with the items in AMSTAR 2.0 and report in lines with the Preferred Reporting Item for Systematic Review and Meta-analysis (PRISMA).

## 2 Methods

### 2.1 Study registration

The protocol of this SR has been registered under number CRD42019127810 on PROSPERO (http://www.crd.york.ac.uk/PROSPERO). This SR will be performed in accordance with PRISMA statement guidelines.

### 2.2 Ethical considerations

No ethical approval and patient consent were required for this protocol of SR since this protocol were based on published studies.

### 2.3 Inclusion criteria

#### 2.3.1 Type of studies

RCTs of Tai Chi for improving balance and reducing falls will be included. There will be no restrictions on language or publication date.

#### 2.3.2 Type of participants

Participants enrolling healthy or unhealthy adults will be included. There will be no restrictions on age, gender, race or nation.

#### 2.3.3 Type of interventions

Studies that used Tai Chi for improving balance and reducing falls will be included. There will be no limit on the duration, frequency or style of Tai Chi.

#### 2.3.4 Type of comparators

The comparative intervention will have to be usual care, other exercises or no treatment.

#### 2.3.5 Outcome measurements

Primary outcomes will be fall-related indicators, including the number of falls, fall rate or other fall-related outcomes. Additional outcomes will include the Berg Balance Scale (BBS) score, standing-walk test, single-legged time or other balance-related measurements.

### 2.4 Exclusion criteria

① Non-RCTs, cross-over RCTs; cluster randomized trials, array studies, reviews, case-control studies; ② Tai Chi combined with other methods; ③ Duplicate or the data cannot be extracted; ④ Full text cannot be obtained through various approaches.

### 2.5 Database and search

The following databases will be searched from inception to March 2019: China Biology Medicine (CBM), China National Knowledge infrastructure (CNKI), Wan Fang Data, the Chinese Science and Technology Periodical Database (VIP), PubMed, EMBASE, Web of Science and The Cochrane Library, using the combination of key words of Tai Chi, fall, balance, randomized controlled trial and RCT. The RCT registration websites (http://www.ClinicalTrial.gov and http://www.chictr.org.cn) will be searched for more related studies. A professional medical librarian will help us to design the terms to retrieve trials enrolling Tai Chi for improving balance and reducing falls. Ambiguous literature will be investigated manually to avoid missing eligible trials. Reference lists of identified publications will also be manually checked. No language or publication date restrictions will be set. The concrete search strategy is shown in appendix 1.

### 2.6 Studies selection

All the retrieved studies will be imported into Endnote (X8) and the duplicated studies will be filtered. Two reviewers will screen the studies by titles and abstracts independently according to the eligibility criteria. The full text of all possibly relevant studies will be downloaded after cross-checked by 2 reviewers. The downloaded studies will be further assessed independently and cross-checked by 2 reviewers. In case of disagreements, a third reviewer will be involved in.

### 2.7 Data extraction

Two reviewers will independently extract data with a pre-defined data extraction form, in which study characteristics (first lead author, publication year and country), participant characteristics (sample size, mean age, health status), methodological characteristics (interventions, comparisons), results (main conclusions, adverse effect) will be included. The authors will be contacted immediately for more information when data reported in RCTs was missing. The data will be cross-checked by 2 reviewers in case of mistakes. As for discrepancy, it will be resolved through team discussion.

### 2.8 Risk of bias assessment

The Cochrane risk of bias tool (www.cochrane-handbook.org.) will be used independently by 2 authors to assess the risk of bias including the following items: random sequence generation, allocation concealment, blind subjects and therapists, blind assessors, incomplete outcome data, selective outcome reporting, and other bias. The risk of bias is categorized as low (meet all criteria)/unclear (trials with insufficient information to judge)/high risk (meet none of the criteria) of bias. In case of disagreements, an agreement will be reached through discussion or a third reviewer will be involved in.

### 2.9 Data analysis

If it is possible to carry out a meta-analysis, Review Manager V5.3 software will be used. The relative risk (RR) will be used to analyze dichotomous outcomes. The mean difference (MD) will be used to analyze continuous outcomes with the same unit. Otherwise, the standardized mean difference (SMD) will be used. The uncertainty will be presented with 95% confidence intervals (95% CI).

We will assess heterogeneity using the I^2^ statistic. Fixed-effects model (FEM) will be used if acceptable heterogeneity is found. Random-effect model (REM) will be used where significant statistical heterogeneity exists. Heterogeneity will be further explored using meta-regression with backward elimination to analyze the associations between treatment effect and the participant characteristics. Results will be described qualitatively in the text when meta-analysis is not possibly carried out.

#### 2.9.1 Dealing with missing data

If the extracted data is missing, the original authors will be contacted for more information. If there were no reply from the original authors, we will try to calculate the data through the available coefficients, the potential impact of these missing data on the results of this SR will be tested in the sensitivity analysis.

#### 2.9.2 Subgroup analysis

To investigate potential heterogeneity across studies, we will conduct subgroup analysis based on age, sex, different styles of Tai Chi, the health status of participants (healthy, stroke, Parkinson disease, knee arthritis, cancer, etc), different places where participants lived (such as nursing home, residential care facilities or community).

#### 2.9.3 Sensitivity analysis

Sensitivity analysis of primary outcomes will be carried out to verify the robustness of the study conclusions by assessing the impact of methodological quality, study design, sample size, and the effect of missing data as well as the analysis methods on the result of this review.

#### 2.9.4 Publication bias

For publication bias, each included study will be assessed according to the CONSORT criteria. The Egger test will be conducted to check whether there is a statistical significance. If the numbers of trials reporting the primary outcomes are over 10, funnel plot will be performed to assess the potential of publication bias of the included studies. If funnel plots are asymmetric, we will try to interpret funnel plot asymmetry.

### 2.10 GRADE

We will evaluate the quality of evidence of outcomes by using the GRADE system. The quality of the index will be evaluated from the following 5 aspects: limitations, inconsistency, indirectness, imprecision, and publication bias.^[42]^ The quality of evidence will be graded as “high”, “moderate”, “low”, or “very low” in accordance with the GRADE rating standards.^[43]^ The results of GRADE including evidence profile (EP) and summary of finding table (SoF) will be generated using GRADE pro software.

## 3 Discussion

As a mind-body exercise, Tai Chi combines Chinese qigong and meditation and consists of a series of movements linked in a continuous sequence, flowing slowly and smoothly from one movement to another that emphasizes weight transfer and movement of the body.^[44]^ Tai Chi needs mental concentration, physical balance and coordination, muscle relaxation, and relaxed breathing, which is regarded as an adjuvant therapy in rehabilitation.

Recently, the beneficial role of Tai Chi for improving balance and reducing falls on healthy or unhealthy people becomes a research hotspot. We did an assessment on the methodological quality of the SRs on Tai Chi for improving balance and reducing falls by using AMSTAR 2.0. The results were shown in Table 1 . 18 SRs were included in our study, 17 SRs were considered critically low quality and 1 SR was low quality. The scores of item 2, item 3, item 7 and item 10 were particularly low. All the included SRs contained the components of PICO, but only 2 SRs reported study protocols in advance. All the SRs had selected study type of RCT or CCT, without explaining specific reasons for selection. Also, no SR provided a complete list of excluded studies with justification and no SR reported the founding sources for the study included. Owing to the flaws of SRs, higher level evidence is needed to verify the effectiveness of Tai Chi for improving balance and reducing falls.

**Table 1:**
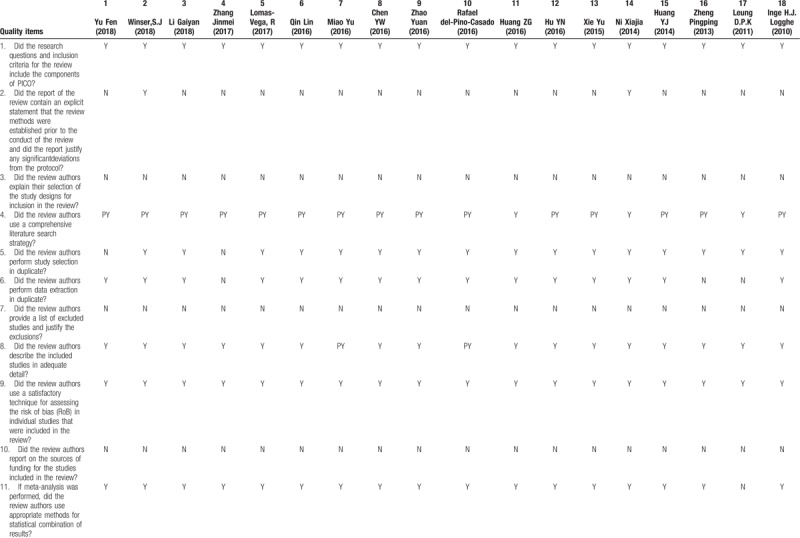
AMSTAR 2.0.

**Table 1 (Continued):**
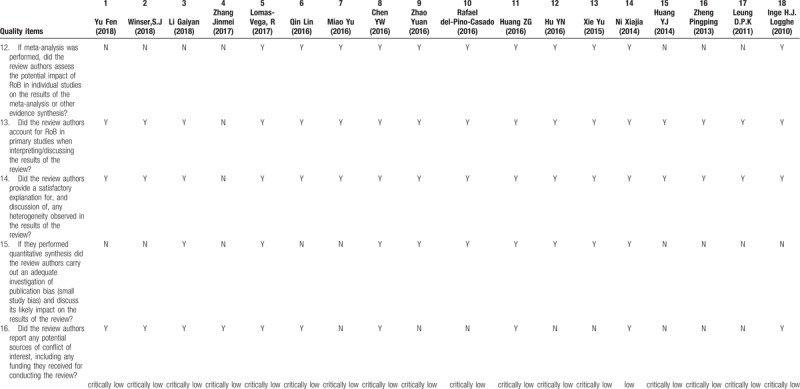
AMSTAR 2.0.

This study aims to investigate the effectiveness and safety of Tai Chi for improving balance and reducing falls on people. The results of this SR will provide more reliable evidence of Tai Chi for improving balance and reducing falls, which may broaden the clinical application of Tai Chi exercise.

The finding of this SR will be disseminated through publication in a peer-reviewed journal. The results of the completed study will be reported according to the Consolidation of Standards for Reporting Trials guidelines^[45]^ and recommendations described in PRISMA statement and the Cochrane Handbook for Intervention Reviews.

## 4 Strengths and limitations

### 4.1 Strengths

This study will be performed and reported strictly according to the standards in the AMSTAR2.0 and PRISMA tools, to reach a high-methodological quality SR. The results of this SR will provide evidence of effectiveness and safety of Tai Chi for improving balance and reducing falls on healthy or unhealthy people. In order to ensure that all relevant studies are included without personal biases, study selection, data extraction, and quality assessment will be performed independently by 2 reviewers.

### 4.2 Limitations

Different types of Tai Chi may cause considerable heterogeneity in this systematic review. A subgroup analysis will be performed based on the type of Tai Chi intervention.

## Author contributions

**Conceptualization:** Juan Li, Rongjiang Jin.

**Data curation:** Yuxi Li, Jing Ye.

**Methodology:** Hui Zheng.

**Project administration:** Lina Xia.

**Writing – original draft:** Dongling Zhong, Qiwei Xiao, Mingxing He.

**Writing – review & editing:** Chi Zhang, Fanrong Liang.

## Supplementary Material

Supplemental Digital Content
