# Supplementary material for: Tai Chi for improving balance and reducing falls: A protocol of systematic review and meta-analysis
Source: Medicine (Baltimore). 2019 Apr 26;98(17):e15225. doi: 10.1097/MD.0000000000015225 (PMC6831220; doi:10.1097/MD.0000000000015225)
Supplement: Supplemental Digital Content [file medi-98-e15225-s001.doc]

**Appendix 1**

**1. Search Strategy for EMBASE:**

#1 'randomization'/exp OR 'randomized controlled trial'/exp OR 'randomized controlled trial (topic)'/exp OR 'controlled clinical trial'/exp OR 'controlled clinical trial (topic)'/exp OR 'clinical trial'/exp OR 'clinical trial (topic)'/exp

#2 tai chi:ab,ti OR taiji*:ab,ti OR qigong:ab,ti OR liuzijue:ab,ti OR wuqinxi:ab,ti OR yijinjing:ab,ti OR baduanjin:ab,ti OR traditional exercise:ab,ti OR chinese traditional exercise:ab,ti OR traditional chinese exercise:ab,ti OR chinese exercise:ab,ti

#3 #1 AND #2

**2.Search Strategy for Medline:**

#1 "Randomized Controlled Trials as Topic"[Mesh] OR "Randomized Controlled Trial" [Publication Type] OR "Controlled Clinical Trials as Topic"[Mesh] OR "Clinical Trials as Topic"[Mesh] OR "Clinical Trial" [Publication Type]

#2 randomized controlled trial[Publication Type]

#3 #1 OR #2

#4  tai chi[Title/Abstract] OR "taiji*"[Title/Abstract] OR qigong[Title/Abstract] OR wuqinxi[Title/Abstract] OR baduanjin[Title/Abstract] OR "traditional exercise"[Title/Abstract] OR traditional chinese medicine[Title/Abstract] OR "chinese traditional exercise" OR "traditional chinese exercise" OR "chinese exercise"

#5 #3 AND #4
